# The Low Conductivity of *Geobacter uraniireducens* Pili Suggests a Diversity of Extracellular Electron Transfer Mechanisms in the Genus *Geobacter*

**DOI:** 10.3389/fmicb.2016.00980

**Published:** 2016-06-28

**Authors:** Yang Tan, Ramesh Y. Adhikari, Nikhil S. Malvankar, Joy E. Ward, Kelly P. Nevin, Trevor L. Woodard, Jessica A. Smith, Oona L. Snoeyenbos-West, Ashley E. Franks, Mark T. Tuominen, Derek R. Lovley

**Affiliations:** ^1^Department of Microbiology, University of Massachusetts Amherst,Amherst, MA, USA; ^2^Department of Physics, University of Massachusetts AmherstAmherst, MA, USA; ^3^Department of Molecular Biophysics and Biochemistry, Microbial Sciences Institute, Yale UniversityNew Haven, CT, USA; ^4^Department of Physiology, Anatomy and Microbiology, La Trobe UniversityMelbourne, VIC, Australia

**Keywords:** electromicrobiology, microbial fuel cells, microbial nanowires, electron transfer, *Geobacter*

## Abstract

Studies on the mechanisms for extracellular electron transfer in *Geobacter* species have primarily focused on *Geobacter sulfurreducens*, but the poor conservation of genes for some electron transfer components within the *Geobacter* genus suggests that there may be a diversity of extracellular electron transport strategies among *Geobacter* species. Examination of the gene sequences for PilA, the type IV pilus monomer, in *Geobacter* species revealed that the PilA sequence of *Geobacter uraniireducens* was much longer than that of *G. sulfurreducens*. This is of interest because it has been proposed that the relatively short PilA sequence of *G. sulfurreducens* is an important feature conferring conductivity to *G. sulfurreducens* pili. In order to investigate the properties of the *G. uraniireducens* pili in more detail, a strain of *G. sulfurreducens* that expressed pili comprised the PilA of *G. uraniireducens* was constructed. This strain, designated strain GUP, produced abundant pili, but generated low current densities and reduced Fe(III) very poorly. At pH 7, the conductivity of the *G. uraniireducens* pili was 3 × 10^-4^ S/cm, much lower than the previously reported 5 × 10^-2^ S/cm conductivity of *G. sulfurreducens* pili at the same pH. Consideration of the likely voltage difference across pili during Fe(III) oxide reduction suggested that *G. sulfurreducens* pili can readily accommodate maximum reported rates of respiration, but that *G. uraniireducens* pili are not sufficiently conductive to be an effective mediator of long-range electron transfer. In contrast to *G. sulfurreducens* and *G. metallireducens*, which require direct contact with Fe(III) oxides in order to reduce them, *G. uraniireducens* reduced Fe(III) oxides occluded within microporous beads, demonstrating that *G. uraniireducens* produces a soluble electron shuttle to facilitate Fe(III) oxide reduction. The results demonstrate that *Geobacter* species may differ substantially in their mechanisms for long-range electron transport and that it is important to have information beyond a phylogenetic affiliation in order to make conclusions about the mechanisms by which *Geobacter* species are transferring electrons to extracellular electron acceptors.

## Introduction

The presence of *Geobacter* species is often equated with processes in which the capacity for long-range electron transfer via electrically conductive pili (e-pili) is an advantageous feature (Lovley et al., 2011). For example, molecular analyses have demonstrated that *Geobacter* species are often among the most abundant microorganisms on anodes harvesting electrons from organic wastes and sediments, as well as in soils and sediments in which Fe(III) reduction is an important process (Kiely et al., 2011; Lovley et al., 2011). Direct interspecies electron transfer (DIET) in anaerobic digesters has been attributed to an abundance and high metabolic activity of *Geobacter* species (Morita et al., 2011; Rotaru et al., 2014b; Shrestha et al., 2014). Long-range electron transport through *Geobacter* anode biofilms, as well as Fe(III) oxide reduction and syntrophy via DIET in *Geobacter* species, have all been linked to long-range electron transport through e-pili (Lovley, 2011; Malvankar and Lovley, 2014).

However, the concept that *Geobacter* species rely on e-pili for long-range electron transport is based on a rather limited dataset from studies primarily conducted with *G. sulfurreducens*. This species has been the focus of most studies because it was the first *Geobacter* species for which a genetic system (Coppi et al., 2001) and genome sequence (Methé et al., 2003) became available and because *G. sulfurreducens* produces high current densities (Nevin et al., 2008; Yi et al., 2009). More limited data are also available for *Geobacter metallireducens* which is also genetically tractable (Tremblay et al., 2012; Shrestha et al., 2013; Smith et al., 2013; Rotaru et al., 2014a,b). Efforts to genetically manipulate other *Geobacter* species have as yet been unsuccessful.

Deletion of the gene for PilA, the type IV pilus monomer in *G. sulfurreducens*, revealed the importance of the pili in Fe(III) oxide reduction (Reguera et al., 2005), current production (Reguera et al., 2006; Nevin et al., 2009), and DIET (Summers et al., 2010). A strain with a genetically modified PilA that yielded poorly conductive pili was also defective in extracellular electron transfer (Vargas et al., 2013), as was a strain of *G. sulfurreducens* that expressed non-conductive *Pseudomonas aeruginosa* pili (Liu et al., 2014). Evidence for the importance of pili in extracellular electron transfer in *G. metallireducens*, which is closely related to *G. sulfurreducens* (Lovley et al., 2011), was specific expression of the pili in *G. metallireducens* when growing on Fe(III) or Mn(IV) oxides (Childers et al., 2002) and the finding that deleting the gene for PilA in *G. metallireducens* inhibited Fe(III) oxide reduction, current production, and DIET (Tremblay et al., 2012; Shrestha et al., 2013; Rotaru et al., 2014a,b).

Consistent with the proposed role of the *G. sulfurreducens* pili in long-range electron transport, chemically fixed pili were conductive across their diameter (Reguera et al., 2005). Networks of unfixed, hydrated pili conducted electrons across a 50 μm non-conducting gap between gold electrodes, suggesting the potential for electron transport along the length of the pili (Malvankar et al., 2011). Charge injected into pili propagated along the length of the pili in a manner similar to carbon nanotubes (Malvankar et al., 2014). Although the c-type cytochrome OmcS was localized on the pili (Leang et al., 2010), the possibility of cytochrome-based electron transport along the length of the pili was ruled out by several lines of evidence, which included the findings that (i) denaturing cytochromes had no impact on conduction of the pili networks (Malvankar et al., 2011); (ii) charge propagated along substantial lengths of the pili that lacked cytochromes (Malvankar et al., 2014); (iii) modifying the pilus structure by replacing aromatic amino acids with alanine yielded pili that were poorly conductive, even though OmcS was properly localized on the pili (Vargas et al., 2013); (iv) *P. aeruginosa* pili expressed in *G. sulfurreducens* were poorly conductive, even though OmcS was properly localized on the pili (Liu et al., 2014); and (v) the cytochromes were spaced too far apart for cytochrome-to-cytochrome electron transport to be feasible (Leang et al., 2010; Malvankar et al., 2012). The conductivity along the length of cytochrome-free sections of individual pili at pH 7 (51 mS/cm) compares favorably with the conductivity of nanowires of similar diameter produced with synthetic conducting organic polymers (Adhikari et al., 2016).

**FIGURE 1:**
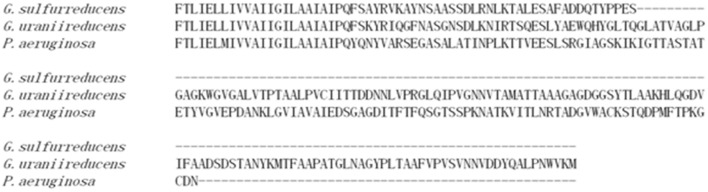
**Alignment of PilA amino acid sequences of *Geobacter sulfurreducens, Geobacter uraniireducens*, and *Pseudomonas aeruginosa***.

*Geobacter sulfurreducens* pili conductivity is attributed to a truncated PilA, which is substantially shorter than the PilA found in most bacteria (Reguera et al., 2005), and permits tighter packing of aromatic amino acids that participate in electron transport (Malvankar et al., 2015). However, not all *Geobacter* species contain a truncated PilA. The PilA of *G. uraniireducens* is much longer (193 amino acids) than the PilA of *G. sulfurreducens* (61 amino acids; **Figure 1**). Unlike *G. metallireducens* (Childers et al., 2002; Shrestha et al., 2013) and *G. sulfurreducens* (Nevin et al., 2009; Shrestha et al., 2013), which highly express *pilA* when growing with an extracellular electron acceptor, *G. uraniireducens* did not upregulate expression of *pilA* when grown on Fe(III) oxide (Holmes et al., 2008). Furthermore, unlike *G. sulfurreducens* and *G. metallireducens*, *G. uraniireducens* did not produce the high current densities that have been attributed to electrically conductive pili, and *G. uraniireduccens* could not participate in DIET (Rotaru et al., 2015). These results suggest that *G. uraniireducens* might not rely on conductive pili for extracellular electron transfer. If so, this would significantly impact on the understanding of the mechanisms long-range electron transport in *Geobacter* species. However, the lack of tools for genetic manipulation of *G. uraniireducens* has limited experimental approaches to evaluate this hypothesis.

There are alternatives to e-pili for some forms of extracellular electron transfer in *Geobacter* species. Studies with *G. sulfurreducens* have demonstrated that cells in direct contact with electrodes do not require e-pili for extracellular electron transfer (Lovley, 2011, 2012). The electrical contact between cells and the anode appears to be made by *c*-type cytochromes, most notably OmcZ (Richter et al., 2009; Inoue et al., 2010, 2011). Outer surface *c*-type cytochromes are also important for the reduction of soluble extracellular electron acceptors (Lovley, 2011), including electron shuttles that may be found in the environment (Voordeckers et al., 2010). Although some microorganisms produce their own electron shuttles to facilitate electron transfer to electrodes or Fe(III) oxides, the *Geobacter* species that have been studied to date do not (Lovley, 2011). *G. uraniireducens* highly expresses a suite of outer-surface *c*-type cytochromes during growth on Fe(III) oxides, suggesting the likely importance of these cytochromes in extracellular electron transfer (Holmes et al., 2008; Aklujkar et al., 2013).

The purpose of this study was to further investigate the possibility for pili-mediated long-range electron transport in *Geobacter* species by evaluating the conductivity of individual *G. uraniireducens* pili. The results demonstrate that *G. uraniireducens* pili are poorly conductive and suggest that *G. uraniireducens* relies on other strategies for extracellular electron transfer.

## Materials and Methods

### Bacterial Strains, Plasmids, and Culture Conditions

All bacterial strains and plasmids used in this study are summarized in Supplementary Table S1. *Geobacter* strains were routinely cultured at 30°C under strict anaerobic conditions (80/20 N_2_–CO_2_) in NBAF medium containing acetate (15 mM) as the electron donor and fumarate (40 mM) as the electron acceptor, as previously described (Coppi et al., 2001). Chemically competent *Escherichia coli* TOP10 (Invitrogen, Grand Island, NY, USA) was used routinely for cloning and cultured at 37°C in lysogeny broth medium (LB medium; Bertani, 1951) with the appropriate antibiotic added when necessary.

### Construction of *G. sulfurreducens* Strain GUP

The *G. sulfurreducens* strain GUP (*G. uraniireducens* pili) was constructed using a previously described approach (Vargas et al., 2013). Primers used for construction of strain GUP are listed in Supplementary Table S2. The three DNA fragments were generated independently by PCR for the construction of the mutant. Primer pair GspilAf/GsupilAr amplified the promoter region of the pilA gene using pPLT174 (Vargas et al., 2013) as the template for the generation of fragment 1. For the generation of fragment 2, primer pair GupilAf/GupilAr amplified Gura_2677 using *G. uraniireducens* RF4 genomic DNA as the template. Primer pair GupilACf/GspilACr amplified 500 bp downstream of the pilA gene using *G. sulfurreducens* genomic DNA as the template for the generation of fragment 3. Three independent fragments for strain GUP were combined via recombinant PCR with primer pair GspilAf/GspilACr as previously described (Liu et al., 2014).

The corresponding recombinant PCR products were digested with *Xho*I and *Apa*I (New England BioLabs, Ipswich, MA, USA) and ligated with the vector pPLT173 (Vargas et al., 2013) using T4 DNA ligase. The plasmid pPLT173 contains 500 bp upstream of the *Geobacter* pilA gene followed by a gentamicin resistance cassette and *Xho*I and *Apa*I restriction site. The final plasmid (pPLT173-GUP) was linearized with *Nco*I (NEB) and electroporated into *G. sulfurreducens* competent cells as previously described (Coppi et al., 2001). Transformants were selected and verified as previously described (Liu et al., 2014).

### Current Production and Fe(III) Oxide Reduction

Current production was determined as previously described (Nevin et al., 2009) in flow-through, two-chambered H-cell systems with acetate (10 mM) as the electron donor and graphite stick anodes (65 cm^2^) poised at 300 mV versus Ag/AgCl as the electron acceptor.

Growth with Fe(III) oxide as the electron acceptor was evaluated as previously described (Vargas et al., 2013) in medium with acetate as the electron donor and poorly crystalline Fe(III) oxide (100 mmol l^-1^) as the electron acceptor (Lovley and Phillips, 1988). Fe(II) production was measured with the ferrozine assay (Lovley and Phillips, 1988). For studies on the need for direct contact for Fe(III) oxide reduction, the poorly crystalline Fe(III) oxide was incorporated into microporous alginate beads (diameter, 5 mm) with a nominal molecular mass cutoff of 12 kDa, as previously described (Nevin and Lovley, 2000). Beads were added to medium to provide Fe (III) at 150 mmol l^-1^. When noted anthraquinone-2,6-disulfonate (AQDS) was added as an electron shuttle at 50 μM. The production of Fe(II) was determined with the ferrozine assay after the beads had been extracted for 12 h in 0.5 N HCl.

### Pili Preparation

*Geobacter sulfurreducens* strain GUP biofilms grown on graphite electrodes as described above were gently scraped from the electrode surface with a plastic spatula and isotonic wash buffer (20.02 mM morpholinepropanesulfonic acid, 4.35 mM NaH_2_PO_4_⋅H_2_O, 1.34 mM KCl, 85.56 mM NaCl, 1.22 mM MgSO_4_⋅7H_2_O, and 0.07 mM CaCl_2_⋅2H_2_O). The cells were collected by centrifugation and re-suspended in 150 mM ethanolamine buffer (pH 10.5). Pili were sheared from the cells with a blender at low speed for 1 min. The cells were removed by centrifugation at 13,000 × *g*. The pili in the supernatant were precipitated with 10% ammonium sulfate overnight and the precipitation was collected with centrifugation at 13,000 × *g* (Brinton et al., 1978). In order to further clean the pili, the precipitation was re-suspended in ethanolamine buffer and then additional debris were removed by centrifugation at 23,000 × *g*. The pili were again precipitated with 10% ammonium sulfate and the precipitation was again collected with centrifugation at 13,000 × *g* (Brinton et al., 1978). The final pili preparation was re-suspended in the ethanolamine buffer and stored at 4°C.

### Transmission Electron Microscopy and Confocal Scanning Laser Microscopy

For the confocal laser scanning microscopy, the anode biofilms were imaged with LIVE/DEAD BacLight viability stain kit from Molecular Probes (Eugene, OR, USA) as previously described (Franks et al., 2010; Nevin et al., 2011). Images were processed and analyzed using the Leica LAS software (Leica). For transmission electron microscopy cells from the anode biofilms were directly placed on copper grids coated with carbon and absorbed for 4 min. The cells were negatively stained with 0.2% uranyl acetate and examined with a JEOL 2000fxTEM at a 200 kV accelerating voltage.

### Pili Dissociation

Pili were suspended in water and dried in a SpeedVac at room temperature. The dried preparations of pili were resuspended in the 20 μL 1% SDS (pH 1.5) and boiled for 5–10 min. The samples were neutralized with 1 N NaOH and 9 μg of protein for each sample was used for the SDS–PAGE analysis. SDS–PAGE analyses were performed using 12.5% (wt/vol) polyacrylamide gels. Proteins were stained with Coomassie brilliant blue R-250.

### Pili Conductivity Measurements

The electrodes were fabricated using nano-imprint lithography (NIL) method on silicon substrate with 1000 nm thick thermally grown oxide. The substrate was cleaned with a Piranha solution (H_2_SO_4_:H_2_O_2_ = 3:1) and a diluted HF solution before patterning. Then 50-nm-thick poly(methyl methacrylate) (PMMA) was spin coated on the substrate followed by a 60 nm thick UV-curable resist. Circuit patterns including 50 nm electrodes separated by 50 nm spacing, microscale fanouts, and contact pads were transferred from a quartz mold to the UV resist using NIL in a homemade imprint chamber. The residual UV-resist layer and the PMMA underlayer were removed in fluorine based reactive ion etching (RIE; CHF_3_/O_2_) followed by oxygen-based RIE. Thin films of 5-nm-thick Titanium (Ti) and 15-nm-thick gold (Au) were then deposited in an electron beam evaporator, followed by a liftoff process in acetone with ultrasonication.

As previously described (Adhikari et al., 2016), a solution (2 μl) of pili in ethanolamine buffer was dropcast on the substrate with electrodes. After letting the pili settle down for about 5 min, the sample was washed for three times with deionized water, which removed salts and left monolayer of pili on the substrate. Pili were localized with atomic force microscopy (AFM) imaging. The pili were then exposed to buffer adjusted to pH 7 with HCl and air dried.

Conductivity measurements were performed as previously described (Adhikari et al., 2016). A Keithley 4200 semiconductor characterization system (SCS) was used for the electrical measurements. The source meter for the two probe measurements was equipped with preamplifiers 4100-PA enabling the system with capacity to measure current signal of up to 100 aA. These SMUs were connected to two terminals of the double-shielded box for low noise measurement. The outer metallic box of the double shielded box acted as Faraday’s cage to protect the signal from electrostatic interference while the inner box acted as guard to prevent leakage current through the circuit during the measurement. A constant potential was applied across the sample, and current response over the time was recorded. The current value for each applied potential was generated by averaging the measured steady state current over time.

Conductivity of a single pilus was calculated as

$$\sigma =\frac{G\cdot l}{A}\,\;\;\;\;\;\;\;\;\;\;\;\;\;\;\;\;\;\;\;\;\;\;\;\;\;\;\;\;\;\;\;\;\left(1\right)$$

where, *G* is the conductance value of a single pili extracted from the linear fit of the current–voltage response of the sample, and *l* is the length of the pili between the electrodes. *A* (= π⋅*d*^2^/4) is the cross-sectional area of the pili calculated from the diameter of the pili measured from AFM images.

In order to account for the multiple pili bridging across the electrodes, we treated them to be equivalent to the multiple resistors in parallel. Assuming that all the pili have same resistance, the equivalent resistance of *n* number of pili across electrodes is *R*_eq_ = *R*/*n*, where *R* is individual resistance of the wire. This implies that the equivalent conductance would be *G*_eq_ = *n⋅G*, where *G* is conductance of an individual pili (Supplementary Equation S1). Therefore, conductance of an individual pili can be derived from the equivalent conductance extracted from the linear fit of the CV graph and dividing the value by number of pili bridging the electrodes as observed in the AFM images.

## Results and Discussion

### *Geobacter uraniireducens* Anode Biofilms

**FIGURE 2:**
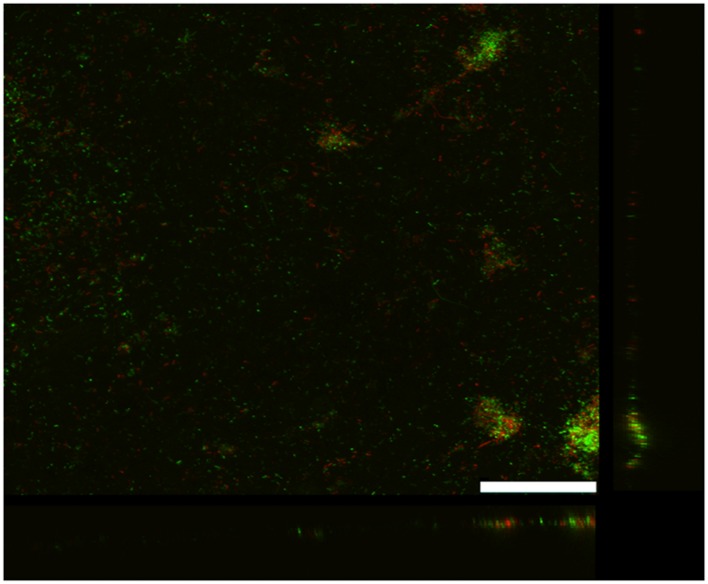
**Confocal scanning laser microscope image of an anode biofilm of *G. uraniireducens* that was producing 0.074 mA/cm^2^ current**. Top-down three-dimensional, lateral side views (right image), and horizontal side views (bottom image) of cells stained with LIVE/DEAD BacLight viability stain. The size bar is 75 microns.

As previously reported (Rotaru et al., 2015), *G. uraniireducens* produces low current densities and this was associated with sparse, thin biofilms (**Figure 2**). Most of the cells were in close contact with the anode surface, suggesting a lack of the electron transport over multiple cell lengths that is associated with the long range electron transfer mediated by e-pili that yields high current densities in *G. sulfurreducens* biofilms.

### *Geobacter uraniireducens*’s Pili Expressed in *G. sulfurreducens*

**FIGURE 3:**
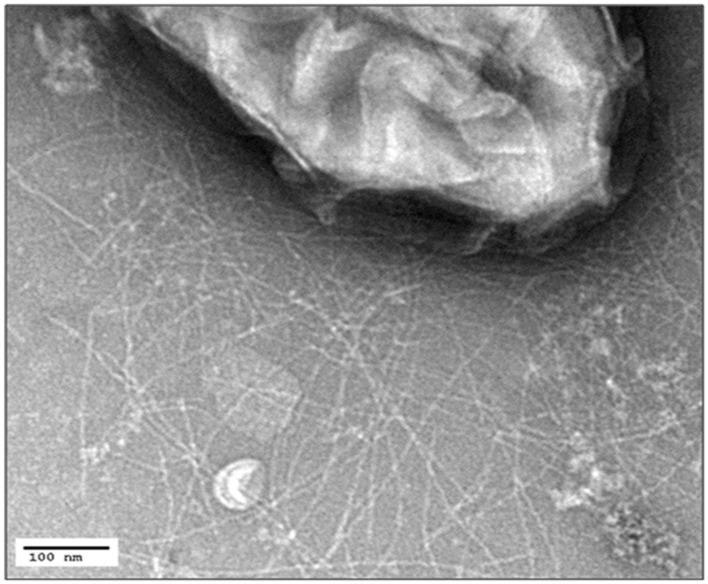
**Transmission electron micrograph of *G. sulfurreducens* strain GUP expressing abundant pili**. The size bar represents 100 nm.

The low biomass obtained on anodes was not sufficient to yield the dense preparations of pili required for conductivity measurements. In an attempt to develop a strain that would produce more *G. uraniireducens* pili, the gene for PilA in *G. sulfurreducens* was replaced with the *pilA* of *G. uraniireducens* with the same gene replacement method previously employed to successfully express other heterologous pili in *G. sulfurreducens* (Vargas et al., 2013; Liu et al., 2014). In medium with acetate as the electron donor and fumarate as the electron acceptor this strain, designated *G. sulfurreducens* strain GUP (*G. uranireducens pili*), grew as well as the control strain (Supplementary Figure S1), which was constructed in the same manner but expressing the *G. sulfurreducens pilA* (Vargas et al., 2013). Strain GUP expressed pili at densities comparable to those previously reported (Vargas et al., 2013) for the control strain (**Figure 3**).

**FIGURE 4:**
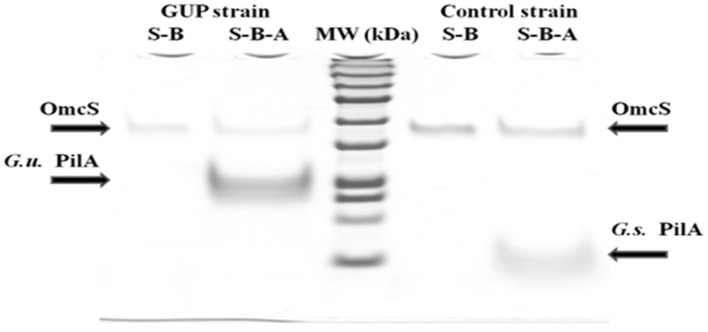
**SDS–PAGE of preparations of strain GUP and control strain pili**. OmcS was detected in controls boiled in SDS (designated S–B) of both types of pili preparations. Pili preparations that were dissociated by boiling in SDS at pH 1.5 (designated S–B–A) contained OmcS as well as the PilA monomer of the expected molecular weight for *G. urannireducens* PilA (20 kDa; designated G.u. PilA) in the GUP strain and *G. sulfurreducens* PilA (6.6 kDa; designated G.s. PilA) in the control strain.

When the pili from the GUP strain were harvested and denatured they yielded a band with a molecular weight consistent with the expected molecular weight molecular weight (20 kDa) of the *G. uraniireducens* PilA, as well as a band for the OmcS c-type cytochrome (**Figure 4**). OmcS has previously been shown to be associated not only with wild-type *G. sulfurreducens* pili (Leang et al., 2010), but also with heterologously expressed pili (Vargas et al., 2013; Liu et al., 2014). As expected based on previous studies (Vargas et al., 2013), pili preparations from the control strain constructed in the same manner but expressing the *G. sulfurreducens* PilA gene sequence, also contained OmcS and the PilA monomer with the molecular weight (6.6 kDa) expected for the *G. sulfurreducens* PilA (**Figure 4**).

### Impact of Heterologously Expressed *G. uraniireducens* Pili on Extracellular Electron Transfer by *G. sulfurreducens*

**FIGURE 5:**
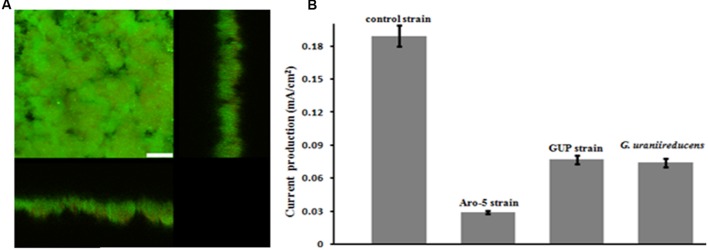
***Geobacter sulfurreducens* strain GUP anode growth and current production**. **(A)** Confocal scanning laser micrographs of graphite anode biofilms of GUP strain producing 0.077 mA/cm^2^ current. Top-down three-dimensional, lateral side views (right image), and horizontal side views (bottom image) of cells stained with LIVE/DEAD BacLight viability stain. The size bar is 25 microns. **(B)** Current production of *G. sulfurreducens* strain GUP compared with previously reported current production levels for the control strain (Vargas et al., 2013), strain Aro-5 (Vargas et al., 2013), and *G. uraniireducens* (Rotaru et al., 2015).

The GUP strain of *G. sulfurreducens* produced substantially thicker biofilms (**Figure 5A**) than *G. uraniireducens* (**Figure 2**). However, current production of the GUP strain was much lower than the *G. sulfurreducens* control strain (**Figure 5B**) with a maximum current density more comparable to that previously reported for *G. uraniireducens* (Rotaru et al., 2015) and strain Aro-5 (Vargas et al., 2013). The formation of thick biofilm with low current densities of the GUP strain is similar to results previously reported for the Aro-5 strain (Vargas et al., 2013).

**FIGURE 6:**
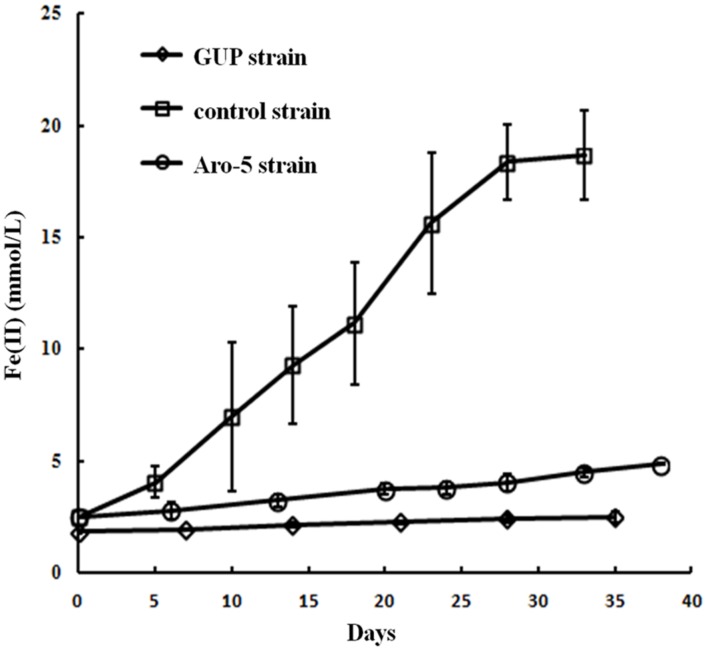
**Fe(III) oxide reduction by the GUP, and previously reported rates of Fe(III) oxide reduction (Vargas et al., 2013) for Aro-5, and control strains of *G. sulfurreducens* strain**. Results are the mean and standard deviation of triplicate determinations.

**FIGURE 7:**
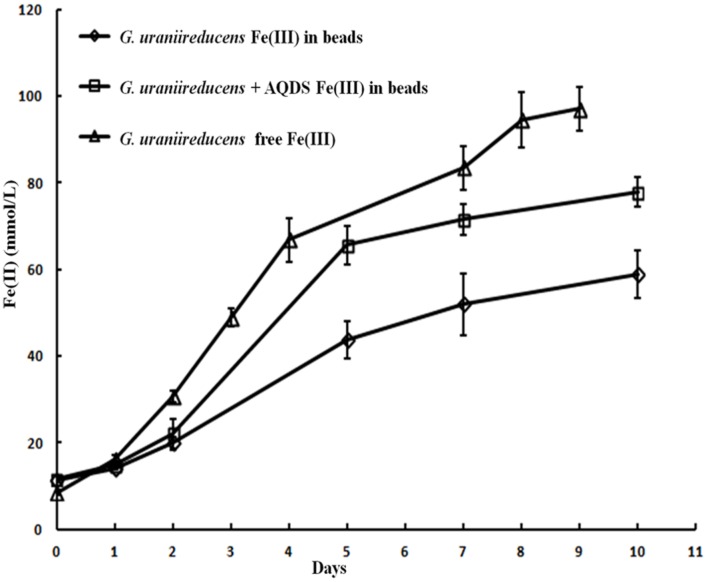
***Geobacter uraniireducens* reduction of Fe(III) oxide sequestered within microporous alginate beads with and without the addition of the electron shuttle AQDS and reduction of Fe(III) oxide not in beads**. Results are the mean and standard deviation of triplicate determinations.

Like the Aro-5 strain, the GUP strain poorly reduced Fe(III) oxide (**Figure 6**). This contrasts with the control strain expressing the *G. sulfurreducens pilA* which readily reduces Fe(III) oxide (Vargas et al., 2013). Although *G. sulfurreducens* GUP strain was not effective in Fe(III) oxide reduction, *G. uraniireducens* is an effective Fe(III) oxide reducer (Shelobolina et al., 2008; Rotaru et al., 2015). Intensively studied Fe(III) oxide-reducing microbes, which based on PilA sequence analysis apparently lack conductive pili, such as *S. oneidensis* and *Geothrix fermentans*, release of compounds that can serve as an electron shuttle between the outer surface of the cell and electron acceptors (Nevin and Lovley, 2002a,b; Bond and Lovley, 2005; Lanthier et al., 2008; Marsili et al., 2008; Von Canstein et al., 2008; Mehta-Kolte and Bond, 2012). Like these organisms, *G. uraniireducens* readily reduced Fe(III) oxide occluded within beads that prevented direct access to the Fe(III) oxide (**Figure 7**), consistent with the release of an electron shuttle that could alleviate the need for conductive pili. In contrast, *G. sulfurreducens* (Smith et al., 2014) and *G. metallireducens* (Nevin and Lovley, 2000) do not reduce Fe(III) oxide within the beads in the absence of an exogenously added shuttle, which is consistent with the proposed reliance on e-pili for long-range electron transport.

### Conductivities of Heterologously Expressed *G. uraniireducens* Pili

**FIGURE 8:**
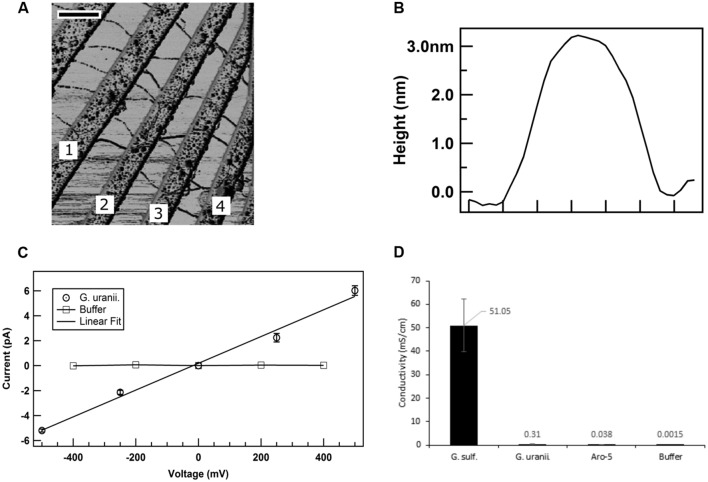
**Conductivity of *G. uraniireducens* pili**. **(A)** Atomic force microscopy image of the *G. uraniireducens* pili bridging electrodes. Electrode pairs 1–2, 2–3, and 3–4 were used for the conductivity measurements. The scale bar represents 500 nm. **(B)** Diameter (height) of the *G. uraniireducens* pili. **(C)** Current–voltage response of the pili. The average value of the current from three measurements are represented as data points while the standard error is represented as the error bar. **(D)** Comparison of the conductivity of pili for *G. uraniireducens* pili and previously reported (Adhikari et al., 2016) conductivities of wild-type *G. sulfurreducens* and Aro-5 pili.

The low current densities and poor Fe(III) oxide reduction by the GUP strain suggested that the *G. uraniireducens* pili expressed in the GUP strain were poorly conductive. In order to directly evaluate the conductivity of the *G. uraniireducens* pili, preparations of pili sheared from the GUP strain were placed on a nanoelectrode array. AFM revealed pili bridging several of the electrodes (**Figure 8A**). The diameter of the pili was 3 nm (**Figure 8B**), comparable to that of the *G. sulfurreducens*. The current–voltage response of pili bridging two electrodes was linear, implying an ohmic effect (**Figure 8C**). The conductivity of the pili at pH 7 was 0.3 ± 0.09 mS/cm (mean ± standard deviation; *n* = 3), which is more than two orders of magnitude lower than the previously reported (Adhikari et al., 2016) conductivity of *G. sulfurreducens* pili at pH 7 (**Figure 8D**).

The electron transfer rate (_Γ_) along the pili can be calculated as

$$\Gamma =\frac{I}{e}=\frac{\sigma \cdot A\cdot V}{e\cdot l}\,\;\;\;\;\;\;\;\;\;\;\;\;\;\;\;\;\;\;\;\;\;\;\;\;\;\;\;\;\;\;\;\;\;\;\;\;\left(2\right)$$

where *I* is the current through the pili at the redox potential difference (*V*) over the pili length (*l*); σ is conductivity; *A* is the cross sectional area of the pili; and *e* is the fundamental electronic charge (1.6 × 10^-19^ coulombs). Thus, a 100-fold decrease in conductivity results in a 100-fold lower rate of electron transfer along the pili under comparable conditions.

Electrical potentials extending within anode biofilms are poorly defined, making it difficult to make an informed estimate of *V*. More information is available for Fe(III) oxide reduction. Although the electron carrier that donates electrons to the *G. sulfurreducens* pili is as yet unknown, a likely candidate is the periplasmic, multi-heme c-type cytochrome PpcA. It is one of the most abundant proteins in *G. sulfurreducens* and is considered to be an important intermediary in electron transport from the inner membrane to outer surface electron transport components (Lloyd et al., 2003). PpcA is a conservative choice for electron transport rate estimates because its mid-point potential of -170 mV is more positive than the mid-point potentials of other potential electron carriers (Lovley et al., 2011).

The midpoint potential of poorly crystalline Fe(III) oxide, the only form of insoluble Fe(III) oxide that *G. sulfurreducens* readily reduces, is 0 mV (Thamdrup, 2000). Therefore, with PpcA as the electron donor and Fe(III) oxide as the electron acceptor *V* is -170 mV. The distance (*l*) between the cells and Fe(III) oxide associated with pili is typically less than 5 μm (Reguera et al., 2005). For wild-type cells, the conductivity of the pili is 5.1 × 10^-2^ S/cm (Adhikari et al., 2016) and the cross-sectional area for a 3 nm diameter pilius is (*A* = πr^2^ = π × (1.5 × 10^-9^ m)^2^ = 7.07 × 10^-18^m^2^). Therefore, the rate of electron flux through an individual e-pilus over 5 μm at a potential difference of -170 mV is estimated (equation 1) to be 7.6 × 10^6^ electrons/s. The maximum potential electron transport for *G. sulfurreducens* has been estimated to be ca. 8 mA/mg protein, or ca. 1.5 × 10^7^ electrons/s per cell (Marsili et al., 2010). Thus, a single conductive pilus could accommodate nearly half the long-range electron transport requirements of *G. sulfurreducens* for Fe(III) oxide reduction at maximum respiration rates. When it is considered that cells typically express more than twenty pili (Reguera et al., 2005; Summers et al., 2010), a single cell’s full complement of pili should be more than sufficient to support electron transport to Fe(III) oxide. However, with the 100-fold lower conductivity of the *G. uraniireducens* pili, rates of electron transport per pili under the same conditions would only be 4.5 × 10^4^ electrons/s, requiring more than 300 pili to support maximum rates of electron transport.

### Implications

The results demonstrate that the pili of *G. uraniireducens* are much less conductive than the pili of *G. sulfurreducens*, reflecting different strategies for long-range electron transport in these species. The lack of a strategy to genetically manipulate *G. uraniireducens* makes it impossible to further examine the function of the *G. uraniireducens* pili with gene deletion studies, but the poor conductivity of the pili and the finding that *G. uraniireducens* reduces Fe(III) oxide with an electron shuttle suggest that long-range electron transport along pili is not an important mechanism for Fe(III) oxide reduction in this organism. *G. uraniireducens* was unable to participate in DIET and produces low current densities (Rotaru et al., 2015), consistent with previous findings that conductive pili are required for DIET and the production of high current densities (Tremblay et al., 2012; Shrestha et al., 2013; Vargas et al., 2013; Rotaru et al., 2014a). The finding that the conductive pili model for long-range electron transport developed from studies with *G. sulfurreducens* does not apply to all *Geobacter* species is an important consideration when interpreting molecular studies of microbial communities involved in extracellular electron transfer. A phylogenetic affiliation with the genus *Geobacter* is not sufficient evidence to assume pili-based long-range electron transfer.

The unique method by which *G. uraniireducens* was isolated was probably an important factor in recovering a *Geobacter* species that does not use conductive pili for long-range electron transport. *G. uraniireducens* was recovered from subsurface sediments directly on solidified medium in which the Fe(III) in the sediment clay fraction served as the electron acceptor. It is unlikely that enough mineral Fe(III) could be incorporated into solidified medium to yield a visible colony if the cells had to be in direct contact with the Fe(III) mineral. However, producing an electron shuttle would permit cells to accesses Fe(III) minerals they could not directly contact. *G. uraniireducens* possess three sets of genes for cytochrome–porin outer membrane complexes that could facilitate extracellular electron transfer to an electron shuttle, or potentially directly to the surface of electrodes or Fe(III) oxides (Aklujkar et al., 2013; Shi et al., 2014). Other cytochromes that are more highly expressed during the reduction of Fe(III) oxides may also have important roles (Holmes et al., 2008; Aklujkar et al., 2013).

The results also suggest that although an electrical conduc tivity can be measured in pili or other filaments it is important to determine whether the conductivity is sufficient to support physiologically relevant rates of electron transfer. For example, the 300 μS/cm conductivity of the *G. uraniireducens* pili is probably too low to support extracellular respiration. Yet *Rhodopseudomonas palustris* filaments of unknown composition implicated in Fe(III) oxide reduction had electrical resistances that correspond to conductivities of only 35–72 μS/cm (Supplementary Equation S2; Venkidusamy et al., 2015). However, the *R. palustris* filaments were chemically fixed and critical point dried prior to the conductivity measurements, which may have altered the filament structure and conductivity. These considerations demonstrate the need for more measurements on the conductivity of microbial filaments to assess the potential for long-range electron transport via filaments in the microbial world.

## Author Contributions

DL, NM, and YT designed the experiments. YT constructed the mutant and performed the experiments for the pili dissociation and Fe(III) oxidizes reduction. RA measured the conductivity of pili. JW prepared for the pili and performed the TEM. KN, OLS, and AF performed the confocal scanning laser microscopy. KN and TW measured the current production. JS conducted the alginate bead assays. YT and DL wrote the initial manuscript draft with revisions contributed from all authors.

## Conflict of Interest Statement

The authors declare that the research was conducted in the absence of any commercial or financial relationships that could be construed as a potential conflict of interest.
