# Supplementary material for: The Low Conductivity of Geobacter uraniireducens Pili Suggests a Diversity of Extracellular Electron Transfer Mechanisms in the Genus Geobacter
Source: Front Microbiol. 2016 Jun 28;7:980. doi: 10.3389/fmicb.2016.00980 (PMC4923279; doi:10.3389/fmicb.2016.00980)
Supplement: Supplementary file 1 [file Data_Sheet_1.DOCX]

Supplementary Material

**The Low Conductivity of *Geobacter uraniireducens*Pili Suggests a Diversity of Extracellular Electron Transfer Mechanisms in the Genus *Geobacter***

Yang Tan, Ramesh Y. Adhikari, Nikhil S. Malvankar, Joy E. Ward, Kelly P. Nevin, Trevor L. Woodard, Jessica A. Smith, Ashley E. Franks, Mark T. Tuominen, Derek R. Lovley*

*** Correspondence:** Derek R. Lovley: Email: dlovley@microbio.umass.edu

| **Table S1 The bacterial strains used in this study.** | | |  |
| --- | --- | --- | --- |
| Strain or plasmid | Relevant characteristic(s) | Source or reference |  |
| Strains |  |  |  |
| *E.coli* |  |  |  |
| Top10 | *recA1 endA1 gyrA96 thi-1 hsdR17* (r_K_^–^ m_K_^+^) *supE44 relA1* 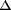*lacU169* | Invitrogen, Carlsbad, CA |  |
| *G.uraniireducens* | Wild type | Lab stock |  |
| *G. sulfurreducens* |  |  |  |
| PCA | Wild type | Lab stock |  |
| GUP strain | PCA *Gm^r^* *G.uraniireducens pilA* | This work | |
| Plasmids |  |  | |
| pPLT174 | pPLT173 carrying P*pilA*; the *pilA* allele; GSU1497 coding sequence; Ap^r^, Gm^r^, Km^r^ | Vargas([Vargas et al., 2013](#_ENREF_1)) | |
| pPLT173 | pCR2.1*gm^r^loxP* carrying the 3’ part of GSU1495 upstream of *gm^r^loxP*; Ap^r^, Gm^r^, Km^r^ | Vargas([Vargas et al. 2013](#_ENREF_1)) | |
| pPLT173 -GUP | pPLT173 carrying *G.uraniireducens pilA* allele; Ap^r^, Gm^r^, Km^r^ | This work | |

Ap^r^, Ampicillin resistance; Gm^r^,Gentamicin resistance; Km^r^, Kanamycin; Tet^r^, Tetracycline; Cm^r^, Chlorophenol resistance .

**Table S2 The primers used in this study**

| Purpose | Primer name | Sequence (5’ to 3’) | Description |
| --- | --- | --- | --- |
| Recombinant  *pil*a gene  construction for GUP strain | GspilAf | AAAAAA*CTCGAG*AGAGGAGCCAGTGACGAAAATC | Amplifies 219 bp upstream of GSU1496 for recombinant PCR |
|  | GsupilAr | ACTTCTCAGTTTGTTTAACATAAGTGTCTCCTTTCTTCTTTT |  |
|  | GupilAf | AAAAGAAGAAAGGAGACACTTATGTTAAACAAACTGAGAAGT | Amplifies Gura_2677 for  recombinant PCR |
|  | GupilAr | CTCCAGTATGTATTTAATCAATTACATTTTTACCCAGTTTGGC |  |
|  | GupilACf | GCCAAACTGGGTAAAAATGTAATTGATTAAATACATACTGGAG | Amplifies 500 bp  downstream of  GSU1496 |
|  | GspilACr | AAAAAA*GGGCCC*ACGAGACTGACCCAATCCAACAAG |  |
| Transformant verification | GupilA2f | ACACTTATGTTAAACAAACTG | Amplifies Gura_2677 |
|  | GupilA2r | TCAATTACATTTTTACCCAGT |  |
|  | GspilA3f | TGGACGAAATCGGAGAAGTGC | Amplifies 68 bp  upstream and 177 bp  downstream of the  construct |
|  | GspilA3r | CAGATGTAAGAGCCGGCAAATAC |  |

**Equation S1**

**Derivation of conductance of an individual pili from multiple pili bridging across two electrodes:**

For Resistors in parallel:

$$\frac{1}{R_{eq}}= \frac{1}{R_{1}}+ \frac{1}{R_{2}}+\ldots+\frac{1}{R_{n}}$$

$$\mathrm{if}, R_{1}= R_{2}= \ldots=R_{n}=R$$

$$\frac{1}{R_{eq}}= \frac{n}{R}\Rightarrow R_{eq}=\frac{R}{n}\Rightarrow G_{eq}=n\cdot G$$

where, n is the number of pili between two electrodes.

**Equation S2**

**Conductivity calculation of *Rhodopseudomonas* *palustris*** ([Venkidusamy et al., 2015](#_ENREF_2))

Resistivity ($\rho$) of pili – 290 -139 Ωm

Hence, conductivity ($\sigma$) = 1/$\rho$= 3.5 – 7.2 mS/m = 35 – 72 μS/cm.

**REFERENCES**

Vargas, M., Malvankar, N. S., Tremblay, P. L., Leang, C., Smith, J. A., Patel, P., et al. (2013). Aromatic amino acids required for pili conductivity and long-range extracellular electron transport in *Geobacter sulfurreducens*. mBio 4:e00105-13. doi:10.1128/mBio.00105-13

Venkidusamy, K., Megharaj, M., Schroder, U., Karouta, F., Mohan, S. V., Naidu, R. (2015). Electron transport through electrically conductive nanofilaments in *Rhodopseudomonas palustris* strain RP2. RSC Adv 5:100790-100798. doi:10.1039/C5RA08742B
